# Phenotype and function of MAIT cells in patients with alveolar echinococcosis

**DOI:** 10.3389/fimmu.2024.1343567

**Published:** 2024-03-14

**Authors:** Jintian Li, Hanyue Zhao, Guodong Lv, Kalibixiati Aimulajiang, Liang Li, Renyong Lin, Tuerganaili Aji

**Affiliations:** ^1^ School of Public Healthy, Xinjiang Medical University, Urumqi, China; ^2^ State Key Laboratory of Pathogenesis, Prevention, and Treatment of Central Asian High Incidence Diseases, Clinical Medical Research Institute, First Affiliated Hospital of Xinjiang Medical University, Urumqi, China; ^3^ Department of Hepatobiliary & Hydatid Disease, The First Affiliated Hospital of Xinjiang Medical University, Urumqi, China

**Keywords:** mucosa-associated invariant T cells, alveolar echinococcosis, parasitic lesion, pro-fibrogenic, serum cytokine

## Abstract

Mucosal-associated invariant T (MAIT) cells are a subpopulation of unconventional T cells widely involved in chronic liver diseases. However, the potential role and regulating factors of MAIT cells in alveolar echinococcosis (AE), a zoonotic parasitic disease by *Echinococcus multilocularis* (*E. multilocularis*) larvae chronically parasitizing liver organs, has not yet been studied. Blood samples (n=29) and liver specimens (n=10) from AE patients were enrolled. The frequency, phenotype, and function of MAIT cells in peripheral blood and liver tissues of AE patients were detected by flow cytometry. The morphology and fibrosis of liver tissue were examined by histopathology and immunohistochemistry. The correlation between peripheral MAIT cell frequency and serologic markers was assessed by collecting clinicopathologic characteristics of AE patients. And the effect of *in vitro* stimulation with *E. multilocularis* antigen (Emp) on MAIT cells. In this study, MAIT cells are decreased in peripheral blood and increased in the close-to-lesion liver tissues, especially in areas of fibrosis. Circulating MAIT exhibited activation and exhaustion phenotypes, and intrahepatic MAIT cells showed increased activation phenotypes with increased IFN-γ and IL-17A, and high expression of CXCR5 chemokine receptor. Furthermore, the frequency of circulating MAIT cells was correlated with the size of the lesions and liver function in patients with AE. After excision of the lesion site, circulating MAIT cells returned to normal levels, and the serum cytokines IL-8, IL-12, and IL-18, associated with MAIT cell activation and apoptosis, were altered. Our results demonstrate the status of MAIT cell distribution, functional phenotype, and migration in peripheral blood and tissues of AE patients, highlighting their potential as biomarkers and therapeutic targets.

## 1 Introduction

Alveolar echinococcosis (AE) is a neglected zoonotic parasitic disease caused by tapeworms *E. multilocularis*, which is mainly parasitic in the liver (1). It is estimated that 91% of the 18,235 new cases of AE annually worldwide occur in China (2, 3), and the pastoral areas of northwestern China are a highly endemic region for *E. multilocularis* (4). The therapeutic approaches of surgical resection and pharmacotherapy remained notable limitations (5): Patients with lesions invading essential blood vessels, serious complications, hepatotoxicity, and adverse drug reactions with long-term medication who urgently need other treatment options to promote a curative prognosis (6).

The regulatory network of immune cells in the liver plays a vital role in maintaining tissue integrity and defending against infection. The *E. multilocularis* infection process stimulates both adaptive and innate immune responses in the host, where macrophages (7), NK cells (8), and T cells (9) regulate the immune response through different mechanisms for host defense (10). It has been shown that treatment based on immune checkpoints PD-1 (11), PD-L1 (12), and TIGIT (13, 14) immunotherapy has potential for the treatment of AE disease but is still not practiced in the clinic. One of the reasons for this is the limited understanding of the composition of the immune system in AE disease.

Mucosal-associated invariant T cells (MAIT) are a distinct population of T cells that express a semi-invariant T cell receptor containing the Vα7.2 segment. These cells are dependent on the non-classical major histocompatibility complex (MHC) related class 1-like molecule, MR1, for the presentation of antigens (15). MAIT are enriched in mucosal sites and comprise 45% of hepatic T cells (16). Capable of producing perforin and granzyme B (GzmB) that directly kill target cells, they secrete interferon γ (IFN-γ), TNF, and IL-17 cytokines to perform rapid effector functions (17). In addition, MAIT cells have been extensively studied in bacterial (18, 19), viral infections (20–22), and tumor diseases (23–25), especially in chronic liver diseases. The collective body of research supports the protective and anti-microbial role of MAIT cells (26–28). Moreover, in malaria-infected parasitic diseases, the frequency of activated MAIT cells increased significantly in a plasmodium falciparum sporozoites pathogens infection dose-dependent manner (29). Our previous study revealed that MAIT cells with the T-cell receptor (TCR) clone TRAV1-2_TRAJ33_TRAC were expanded in AE patients’ peripheral blood and liver tissues and enriched in the liver tissues by single-cell RNA and TCR sequencing (30). However, the phenotype and function of MAIT cells and the correlation with clinicopathologic features are unknown.

In this study, we indicated that reduced MAIT cells in the peripheral blood of AE patients correlate with liver injury and lesion size due to *E. multilocularis* infection. Furthermore, the MAIT cell frequency in the peripheral blood return to control values after surgery. In addition, we revealed that MAIT cells aggregated in liver tissue close to lesions exerted potential anti-AE effects through pro-inflammatory and pro-fibrotic functions. These results extend our insights into the frequency and functional status of MAIT cells in *E. multilocularis* infection and suggest therapeutic targets based on MAIT cells for patients with AE.

## 2 Materials and methods

### 2.1 Human blood samples

Blood samples of 29 patients with *E. multilocularis* infection and 25 healthy donors (HDs) age- and sex-matched were collected at the First Affiliated Hospital of Xinjiang Medical University from 2022 to 2023. Fibrosis stratification was predicted using the aspartate transaminase (AST) -to-platelet ratio index (APRI) and fibrosis-4 interferon-gamma (FIB-4) indexes (22). Patients’ characteristics are listed in **Table 1**. Specimens were collected from AE patients with no co-infections of other types of parasitic diseases, and preoperative (PreOp) blood was collected from AE patients within 0-3 days after admission, and postoperative (PostOp) blood was collected within 7 days when patients were free from other PostOp infections. The study protocol was approved by the ethics committee of the First Affiliated Hospital of Xinjiang Medical University (20211015–53), and written informed consent was obtained from each subject by the Declaration of Helsinki (1975) of the World Medical Association.

**Table 1:** Baseline characteristics of the patients.

Samples	HDs	AE	*p*
**No.**	25	29	–
**Sex (F/M)**	13/12	14/15	0.790
**Age (Year)**	43.52 ± 13.20	44.72 ± 11.42	0.724
**lymphocyte (%)**	32.79 ± 5.62	21.60 ± 7.71	**1.06E-07**
**Monocyte (%)**	7.12 ± 1.66	7.39 ± 2.38	0.624
**AST (U/L)**	26.07 (23.89, 27.33)	38.75 (27.83, 83.07)	**9.80E-05**
**ALT (U/L)**	20.00 (17.50, 27.00)	38.00 (18.50, 74.00)	**0.007**
**ALP (U/L)**	70.02 ± 19.49	188.20 (124.42, 475.27)	**4.87E-08**
**TBIL (μM)**	13.94 ± 3.64	14.00 (8.70, 34.09)	**0.028**
**IBIL (μM)**	4.70 (2.94, 9.86)	6.64 (2.50, 20.03)	0.109
**GGT(U/L)**	20.59 (16.00, 29.12)	90.77 (45.70, 222.49)	**6.52E-08**
**Total protein (g/L)**	77.87 ± 4.36	83.04 ± 10.52	**0.001**
**Globulin (g/L)**	34.14 ± 3.35	46.64 ± 9.92	**3.21E-08**

Dashed line indicates not available. Normal distribution data are shown as mean ± SEM, non-normally distributed data are shown as median (IQR). The bold values is p < 0.05.

### 2.2 AE patient’s liver samples

Liver specimens were collected during hepatic resection or liver transplantation from 10 AE patients. Specimens were divided into two parts: one part of the liver tissue is close to the parasite lesion by about 0.5 cm, named close liver tissue (CLT), and another part is distant from the parasite lesion by at least 2 cm, named distant liver tissue (DLT), which were frozen and formalin-fixed rapidly after resection, and fresh liver tissues extracted and separated tissue mononuclear cells. For all cases, patients’ characteristics are listed in **Supplementary Table 1**. All patients signed an informed consent form, and the study was approved by the First Affiliated Hospital ethics committee of Xinjiang Medical University (20211015–53).

### 2.3 Flow cytometry

As previously described, fresh liver tissue specimens were dissociated into single-cell suspensions for flow cytometry analysis (13). The fresh single-cell suspension and peripheral blood mononuclear cells (PBMCs) used for the analysis of the surface phenotype of MAIT cells and were stimulated with phorbol myristate acetate (PMA; 25ng/mL) for 4h at 37°C in RPMI-1640 medium supplemented with 10% fetal bovine serum (Gibco), followed by surface staining, fixation/permeabilization for detecting IFN-γ, GzmB, and IL-17A cytokine production, and a minimum of 150,000 cells per sample were acquired using the Beckman DXflex (Beckman, USA) flow cytometer and analyzed by FlowJo software (version 10, USA).

The fluorescent-linked antibodies were as follows: CD3 (HIT3a), CD161 (HP-3G10), Vα7.2 (3C10), and CXCR5 (J252D4) antibodies were obtained from BioLegend, France. The CD4 (SK3), CD8 (SK1 and RPA-T8), CD69 (FN50), CD28 (CD28.2), PD-1 (EH12.1), CCR6 (11A9), IL-17A (SCPL1362), GzmB (GB11) and IFN-γ (B27) antibodies were obtained from BD Biosciences, California. The lymphocytes were gated on a population of CD3^+^CD161^+^Vα7.2^+^ T cells (**Supplementary Figure 1**), defined as MAIT cells as previously reported (31). The PE-conjugated 5-OP-RU-loaded MR1 tetramers were used to analyze the surface expression of MR1 by flow cytometry as per the manufacturer’s instructions (31) (**Supplementary Figure 1**). For the MAIT cell frequency and cell surface receptor assays in peripheral blood and liver tissue from AE patients, at least 100,000 cells per sample were collected for analysis using a Beckman DXflex (Beckman, USA) flow cytometer and analyzed using FlowJo software (version 10, USA). Due to the limited availability of collected peripheral blood and tissue samples, the extracted mononuclear lymphocytes were limited, and the MAIT cell frequency was preferentially detected. The gating strategies of surface receptor and cytokine are shown in **Supplementary Figure 2**.

### 2.4 Assessment of short-term *E. multilocularis* protein stimuli *in vitro*

Peripheral blood was collected from 3 HDs per experiment, and PBMC were isolated by the Ficoll-Paque (Solarbio, china), as previously described (13). The anti-CD3 (1 µg/mL) and anti-CD28 (0.5 µg/mL) antibodies diluted in RPMI-1640 medium (Gibco, USA) and supplement 10% fetal calf serum (Gibco, USA) and co-culture with 1*10^5^ PBMCs per well to activate before the co-cultures were exposed to 5 µg/µL and 10 µg/µL Emp for 24h at 37°C, 5% CO2, followed by flow cytometric analysis. DMSO group without Emp were used as controls.

### 2.5 Immunohistochemical and histomorphological staining

The hematoxylin and eosin (HE), Sirius red, and Masson stains were performed on 4 μm-thick serial tissue sections of the DLT and CLT from patients with AE, and immunohistochemical staining with a-SMA antibody (1:500, Abcam, Britain). Whole sections were scanned by KF-PRO-400-HI (KFBIO, China). The positive areas of each tissue section were quantified using Image J by three random fields.

OCT-embedded tissue specimens were incubated overnight at 4°C with anti-CD161 (1:40, Abcam, Britain) and anti-PD-1 (1:50, Abcam, Britain) antibodies in PBS containing 1% BSA and 0.2% Triton X-100. The slides were incubated at room temperature for 1h before adding a secondary antibody, followed by Alexa Fluor^®^ 647 and Alexa Fluor^®^ 488. Nuclear was counterstained by DAPI (1:20, KeyGEN BioTECH, China), and images were obtained by Olympus VS200 confocal microscopy (Leica, Germany).

### 2.6 Serum cytokine assay

The blood samples of pre- and postoperative AE patients, HDs were collected, and centrifuged at 4°C, 3500 rpm, for 15 min to obtain serum. The level of serum cytokines (IL-6, IL-8, IL-12p70, IL-17A, IL-18, IL-33, and IFN-γ) detected by LEGENDplex™ Human Inflammation Panel 1 kit (BioLegend, USA) on flow cytometers, according to manufacturer’s instructions.

### 2.7 Statistical analyses

Statistical calculations were analyzed by GraphPad Prism software (version 8, USA) and SPSS software (version 26, USA). Data with normal distribution and equal variance were analyzed by the student t-test or Analysis of variance (ANOVA) for comparisons of groups. The Mann-Whitney U test or Wilcoxon’s matched-pairs signed-rank test was performed for non-normally distributed variables. The Spearman test was employed for correlation analysis.

## 3 Results

### 3.1 Characteristics of patients

The clinical characteristics of the subjects are summarized in **Table 1.** The age, sex, percentage of monocyte, total bilirubin (TBIL), and globulin between patients with AE and HDs are not significantly different. The alanine aminotransferase (ALT), AST, Indirect Bilirubin (IBIL), γ-glutamyl transferase (GGT), and alkaline phosphatase (ALP) in patients with AE have significantly different (*p*<0.05).

### 3.2 Circulating MAIT cells are exhausted and decreased cytotoxic efficacy in AE patients

**Figure 1:**
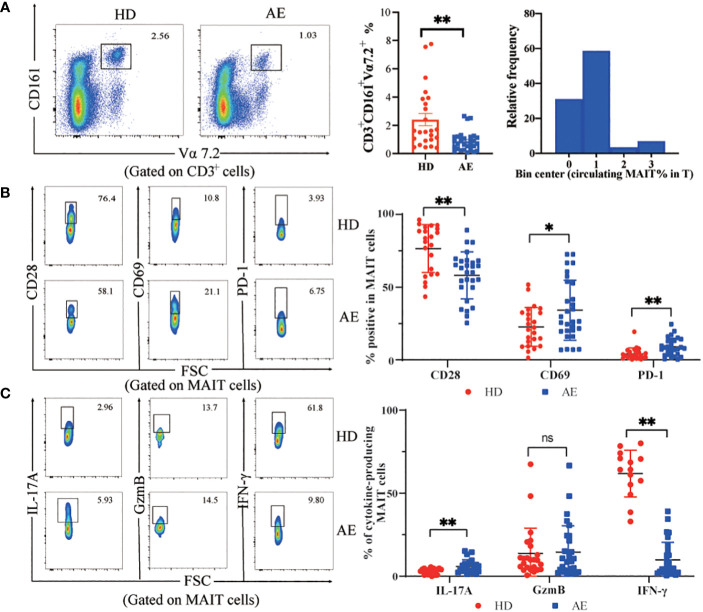
Frequency and functions of circulating MAIT cells are impaired in AE. **(A)** Representative dot plot showing reduction of CD161^+^Vα7.2^+^ double positive (MAIT) cell population in peripheral blood and summary data from AE patients (n = 29), as compared to that in HDs (n = 25), and frequency distribution of CD3^+^CD161^+^Vα7.2^+^ MAIT cell within peripheral blood T cells in AE patients. **(B)** Representative dot plots and data of increased expression of the MAIT cell surface activating receptors CD28 and CD69 with the inhibitory receptor PD-1 from HDs (n = 22-24) and AE patients (n = 28-29). **(C)** Representative dot plots and cumulative data of cytokine profiles of MAIT cells in PBMCs from AE patients (n = 23-29) and HDs (n = 14-24). Statistical analysis was performed using Mann-Whitney or Kruskal-Wallis. ns *p* > 0.05; *p < 0.01; **p < 0.05.

In peripheral blood, the percentage of MAIT cells was lower in AE patients than in HDs, and the relative frequency of MAIT cells in the AE patients is mainly clustered around 1% (**Figure 1**). Circulating MAIT cells from AE patients displayed an activated phenotype, characterized by higher percentage of CD69^+^MAIT cells as compared to HDs (**Figure 1**). The IL-17^+^ MAIT cells from AE patients were higher than HDs. In contrast, the IFN-γ^+^ MAIT cells from AE patients were lower than HDs. The GzmB was no different between the two groups (**Figure 1**). Furthermore, the mean fluorescence intensity (MFI) of cell surface receptors and cytokines was consistent with the percentage statistics (**Supplementary Figure 1**). Altogether, the percentage of circulating MAIT cells is reduced in AE patients, which may be related to activation-induced exhaustion due to persistently high expression of the activation receptor CD69 and the immunosuppressive receptor PD-1 on the surface of MAIT cells in the peripheral blood of AE patients.

### 3.3 Intrahepatic MAIT cells maintain cytotoxic efficacy and are associated with the degree of fibrosis in AE patients

**Figure 2:**
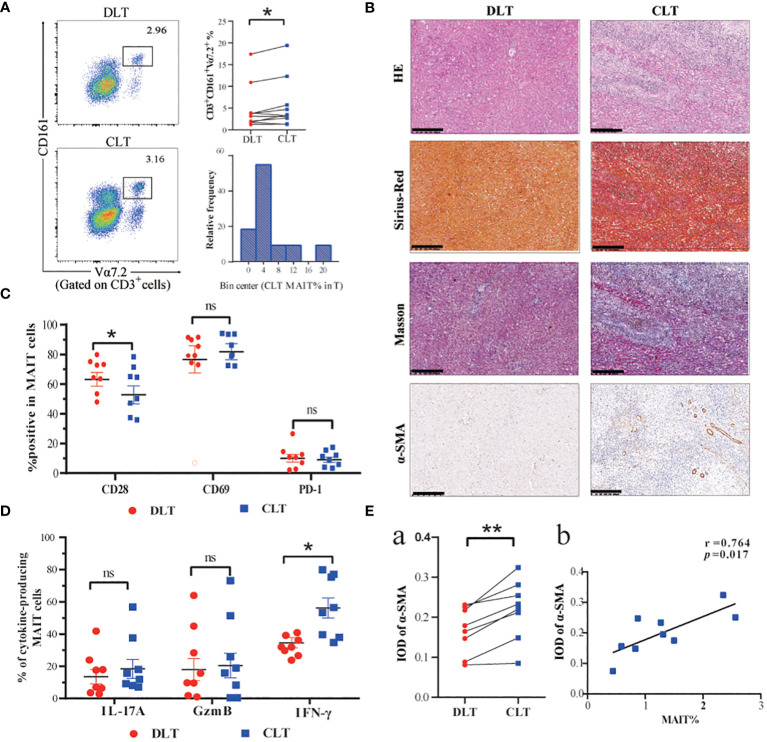
Frequency and functions of hepatic MAIT cells are impaired in AE patients. **(A)** Representative dot plots and cumulative data of MAIT cells in mononuclear cells isolated from CLT and DLT (n=10), and frequency distribution of CD3^+^CD161^+^Vα7.2^+^ MAIT cell within CLT T cells in AE patients. **(B)** Pathologic section staining of CLT and DLT in AE patients (Scale bar = 200 μm). **(C)** Comparison of CD28, CD69, and PD-1 expression in hepatic MAIT cells in CLT and DLT (n = 8). **(D)** Cumulative data of cytokine profiles of hepatic MAIT cells in CLT and DLT (n = 8). **(E)** Statistical and correlation plots of Integral optical density (IOD) values of a-SMA in CLT and DLT. Statistical analyses were performed using paired Wilcoxon tests (a, c, d). ns *p* > 0.05; *p < 0.01; **p < 0.05.

To investigate the phenotype and function of MAIT cells in the livers of patients infected with *E. multilocularis*, we collected close and distal liver tissues from lesions in patients undergoing hepatectomy. The percentage of MAIT cells was higher in the CLT than that DLT, and the relative frequency of MAIT cells in the CLT is mainly clustered around 4% (**Figure 2**). The CLT of AE patients had more inflammatory cell infiltration and higher degree of fibrosis than the DLT (**Figure 2**), moreover, the IOD value of α-SMA in CLT was higher than that of DLT, and notably, the number of MAIT cells was positively correlated with a-SMA (**Figure 2**). In contrast, fibrosis stage, predicted by APRI and FIB-4 index, increasing with decreasing frequency of MAIT cells in the blood (**Supplementary Figure 2**). CD28, T-cell co-stimulatory signal, was fewer in CLT than in DLT, suggesting that MAIT cells in CLT had a diminished stress response to antigens, and surface receptors CD69 and PD-1 were not significantly different between the two groups (**Figure 2**). Co-expression of PD-1 and CD161 was higher in CLT than DLT of AE patients (**Supplementary Figure 3**). Furthermore, in terms of the ability of hepatic MAIT cells to secrete cytotoxic factors, the ability of MAIT cells to secrete IFN-γ was enhanced in the CLT with statistical significance. The levels of secreted IL-17 and GzmB were higher than those of DLT, but not statistically significant (**Figure 2**). The mean fluorescence intensity (MFI) of cell surface receptors and cytokines was consistent with the percentage statistics (**Supplementary Figure 3**).

### 3.4 Intrahepatic MAIT cell frequency correlates with CXCR5 expression and shows greater effector potency than circulating counterparts

**Figure 3:**
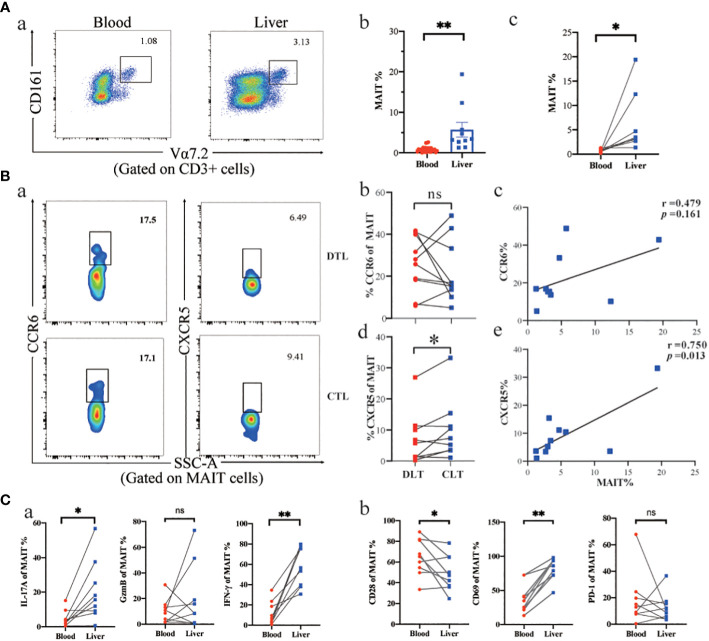
Phenotype and function of MAIT cells in AE patients’ peripheral blood and liver tissue. **(A)** Representative dot plots and cumulative data of peripheral blood versus intrahepatic MAIT cells in AE patients (unpair: blood n = 29, liver n= 9; pair: n = 8). **(B)** Intrahepatic MAIT cells expressing CCR6 and CXCR5 chemokine receptors in CLT and DLT, and correlation between intrahepatic MAIT frequency and the percentage of MAIT cells for CCR6 (n = 10) and CXCR5 (n = 10) in AE patients. **(C)** Cytokine and cell surface receptor of MAIT cells in peripheral blood versus liver tissue in AE patients (n = 8). ns p > 0.05; *p < 0.01; **p < 0.05.

To better understand the role of MAIT cells in peripheral blood and liver tissue, we first described the frequency and phenotype of MAIT cells in peripheral blood and liver tissues of AE patients and related their frequency changes to chemokine receptor expression. The percentage of MAIT cells in the CLT was higher in AE patients than in peripheral blood (**Figure 3**). Moreover, the MAIT cells highly expressed CXCR5 and CCR6 chemokine receptors in CLT compared to the DLT, suggesting that MAIT cells may have the tissue-homing ability during *E. multilocularis* infections. Notably, the expression of CXCR5 in CLT and DLT was significantly different (**Figure 3**-b, d). In addition, there was a significant positive correlation between intrahepatic MAIT cell frequency and their expression of CXCR5 and a tendency for CCR6 (**Figure 3**-c, e). The ability of MAIT cells to secrete IFN-γ and IL-17A cytokines in CLT was higher than in peripheral blood, but GzmB had no significant difference between two groups (**Figure 3**). Next, we investigated the phenotype of circulating and CLT MAIT cells in AE patients. CD28 was decreased in the CLT, and the proliferation and differentiation capacity of naive T cells in AE patients may be weakened. The MAIT cells in CLT highly expressing CD69 receptors, demonstrated that MAIT cells were activated by the parasitic pathogens in liver tissue during infection. However, the PD-1 receptor, which indicates T-cell exhaustion, was not significantly different between the two groups. The results indicated that MAIT cells may be recruited in the CLT by elevated levels of CXCR5 and showed more active phenotype and pro-inflammatory function than circulating MAIT cells.

### 3.5 Decreased frequency of circulating MAIT cells correlates with liver injury caused by long-term *E.multilocularis* parasitism

**Figure 4:**
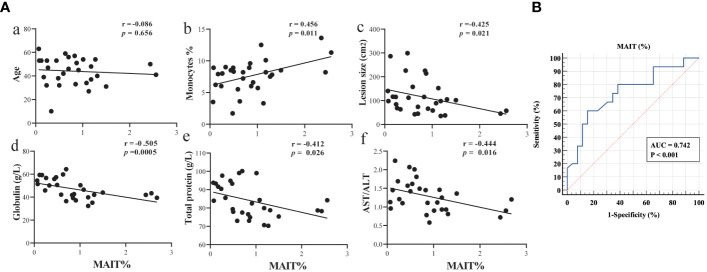
Circulating MAIT cell frequency correlated with liver function in AE patients. **(A)** Spearman correlation between circulating MAIT cell frequency in AE patients and levels of age, percent of monocytes, lesion size, globulin, total protein, and AST/ALT (n = 29); **(B)** ROC curve analysis of the diagnostic value of MAIT cell frequency in AE patients.

To assess the clinical significance of circulating MAIT cells in AE patients, we performed Spearman’s correlation analysis. Based on changes in clinical parameters presented in **Table 1**, we gained further insights regarding age, monocytes, lesion size, globulin, total protein and AST/ALT, and the correlation of MAIT cell frequency with the percentage of lymphocyte, TBIL, IBIL, GGT, AST, and ALT in **Supplementary Figure 4**. Notably, MAIT cell frequency has no correlation with age in patients with AE, showed a significant positive correlation with monocytes, and a significant negative correlation with the size of liver parasitic lesions, globulin, total protein, and AST/ALT (**Figure 4**). To assess the diagnostic ability of circulating MAIT cell frequency as it relates to validated markers of liver function, we constructed a receiver operating characteristic curve (ROC). We evaluated the diagnostic value of MAIT cells for AE and determined the area under the curve, sensitivity, specificity, and Youden’s J statistic to be 0.742, 60.00%, 84.62%, and 0.4462, respectively (**Figure 4**). Taken together, our data reveal a negative correlation between circulating MAIT cell frequency and liver function, and support the notion that MAIT can be a biomarker to evaluate parasite infection of liver injury.

### 3.6 Changes in cytokine profiles and MAIT cytotoxic capacity after surgery in AE patients

**Figure 5:**
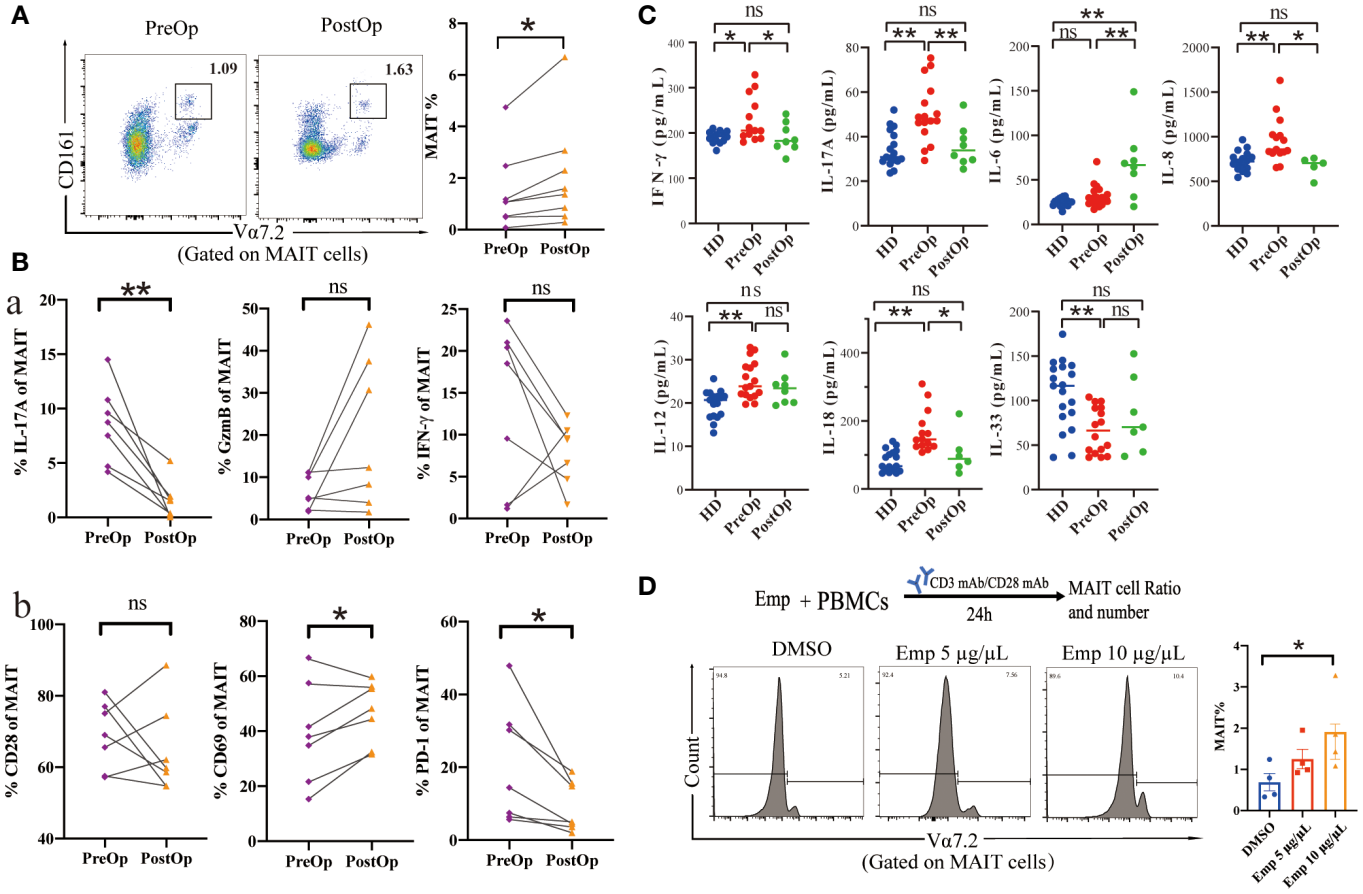
Phenotype and function of circulating MAIT cells in pre- and postoperative AE patients. **(A)** Representative dot plots of pre-and postoperative MAIT cells (n=8). **(B)** Percentage of receptors expression and intracellular cytokine staining of MAIT cells from pre-and postoperative AE patients (n=7). **(C)** Cytokine concentrations in pre-and postoperative AE patients (HDs: n =14-19; PreOp: n =14-19; PostOp: n=5-8). **(D)** Co-culture system of PBMC from HDs incubated with anti-CD3 and anti-CD28 antibodies and the frequency of MAIT cells stimulated by Emp for 24 hours, all data are representative of four independent experiments. Statistical analyses were performed using paired Wilcoxon tests **(A, B)**, ANOVA tests, and a two-sample t-test **(C, D)**. ns *p* > 0.05; *p < 0.01; **p < 0.05.

We investigated the phenotype and effector function of circulating MAIT cells in patients with AE after surgical removal of parasitic lesions. The percentage of circulating MAIT cells in PostOp blood was increased after surgical removal of parasitic lesions compared to PerOp (**Figure 5**). Enhanced activation of circulating MAIT cells in PostOp AE patients was indicated by increased numbers of CD69^+^MAIT (dot plot in **Supplementary Figure 5**). Moreover, the PD-1^+^MAIT and CD28^+^MAIT cells between PostOp and PreOp AE patients have no significantly different (**Figure 5B**). As T cell activation is associated with a change in effector function, compared with PreOp AE patients, IL-17A production was decreased in MAIT cells of PostOp AE patients. The MAIT cells produced IFN-γ and GzmB not significantly different between PostOp and PreOp AE patients (**Figure 5A**).

While the role of MAIT cells in infections has been widely focused, MAIT cells are also regulated by inflammatory cytokines in non-microbial diseases as well (15), we further explored the Preop and Postop changes of inflammatory cytokines in the serum of AE patients. The pro-inflammatory factors IFN-γ, and IL-17A of PostOp serum of AE patients were reduced with the PreOp and were not significantly different with HDs (*p*>0.05). Moreover, the pro-inflammatory factor IL-6 was increased in PostOp patients (**Figure 5**). IL-33, which is involved in the regulation of Th2-type immune and inflammatory responses, was reduced in the serum of PreOp patients compared with HDs, and was not significantly different with PostOp patients. Levels of IL-8, IL-12, and IL-18 cytokines associated with MAIT cell activation and apoptosis were significantly increased in PreOp patients compared with HDs (*p*<0.01), decreased PostOp compared with PreOp patients, and were not significantly different between PostOp patients and HDs (*p*>0.05). In addition, we found that the percentage of MAIT cell significantly increased with Emp concentration in a dose-dependent compared with the DMSO group, implying that MAIT cells are capable of responding in an early phase of infection.

## 4 Discussion

By escaping the host’s immune defenses, *E. multilocularis* proliferated asexually in the liver for long periods triggering an intense immune infiltration around the parasitic lesion, causing changes in the AE patient’s immune environment (32). This study is the first comprehensive analysis of the phenotype, cytokine secretion, and cytotoxicity of MAIT cells during chronic *E. multilocularis* infection from peripheral blood and liver tissue in AE patients.

We initially determined that the frequency of MAIT cells was severely reduced in the circulation of AE patients and displayed an exhausted phenotype. Various factors may contribute to MAIT cell depletion, including age (33), gender (34), redistribution (16), and activation-induced cell death (35). Our results showed that the frequency of MAIT cells in the peripheral blood of AE patients did not correlate with gender and age. Moreover, cellular exhaustion due to high expression of PD-1 and CD69 in circulating MAIT cells of AE patients may contribute to the loss of MAIT cells. In addition, both IL-12 and IL-18 can induce MAIT cell activation and death *in vitro* (36), and they were significantly increased in PreOp but not significantly different in PostOp AE patients compared with HDs, suggesting a possible mechanism for the severe loss of MAIT cells in AE patients. Notably, the reduced peripheral circulating MAIT cells in AE patients may be accumulated into the CLT, and the MAIT cells in the CLT showed enhanced activation capacity and cytotoxicity.

As previously reported MAIT cells proliferation *in situ* in infected organs (37). Tissue-resident molecules CXCR5 and CCR6 are expressed on the surface of MAIT cells, and stimulation by *E. multilocularis* infection causes MAIT cells migrate from the bloods to the site of infection. In particular, CXCR5 expression was positively correlated with the frequency of MAIT cells in CLT. The CXCR5 is a characteristic marker of T cells and mediates T-B cell interactions through CXCL13 chemokines (38, 39). During chronic HBV infection, high intrahepatic expression of CXCL13 promotes CXCR5^+^CD8^+^ T cell aggregation, exerting an immune control effect against HBV infection (40). MAIT cells migrate to the site of infection under chemokine guidance, where they contribute to pathogen clearance and immunoregulation.

Aggregation of MAIT cells in the CLT may be involved in the pathology of hepatic fibrosis caused by *E. multilocularis* infection. The liver fibrosis of AE patients is due to the stimulation of immune cells by *E. multilocularis* to form granulomas around parasitic vesicles, which are pathologically damaged by highly cross-linked collagen (41, 42). MAIT cells were found to accumulate in hepatic fibrotic septa in patients with alcoholic or nonalcoholic fatty liver-associated cirrhosis, and they have the ability to alter the fibrotic properties of hepatic myofibroblasts and exert a pro-fibrotic effect (31, 43). Also, the MAIT cells contribute to the development of hepatic stellate cell-mediated hepatic fibrosis may be a cellular and molecular pathway for fibrosis development in patients with autoimmune liver disease (44). Our results show that the circulating MAIT cells frequency in AE patients tended to decrease with the fibrosis stage, whereas the intrahepatic MAIT cells frequency was positively correlated with the expression of fibrosis indicator α-SMA. As speculated by previous studies, it is possible that MAIT cells accumulate in the liver tissue around the parasite lesion before they are depleted in peripheral blood, and exhibit pro-fibrogenic role to limit *E. multilocularis* larval growth.

MAIT cells are a key component of the immune system and are considered an important bridge between innate and adaptive immunity (15). Activation of innate and adaptive immunity plays a vital role in the parasitic process of *E. multilocularis* (12). The development of the MR1 tetramer tool loaded with 5-OP-RU promotes the previous studies of MAIT cells that relied on specific cell surface markers such as CD3, Va7.2, and CD161 (15). Here, the CD3, CD161, and Vα7.2 containing and the MR 1 tetramer labeling MAIT cells displayed similar percentages in the PBMCs of AE patients (**Supplementary Figure 1**). MAIT cells recognize vitamin B-based metabolite antigens presented by the MHC Class I related-1 protein, MR1, and MR1-deficient mice model lacking MAIT cells affects disease susceptibility (45). MR1-deficient mice showed impaired ability to response to infection with *E. coli* or *M. abscessus* (46), and to T cell respond to infection with bovis bacillus Calmette-Guérin before the full induction of adaptive immunity (47). Moreover, adoptive transfer of MAIT cells into hosts rescues immunodeficient mice lacking of T cells or NK cells from lethal *Legionella* infection (18). Additionally, the frequency of MAIT cells in the blood of patients correlates with the severity of ICU-acquired infections (48), and the frequency of intrahepatic MAIT cells in patients with hepatocellular carcinoma (24) and cholangiocarcinoma (23) was associated with overall survival. Thus, MAIT cells have the potential to be used as therapeutic targets and prognostic markers in immunological studies and clinical applications.

MAIT cells respond to a wide array of pathogens through diverse activation mechanisms, including both TCR-dependent and independent pathways, and play a crucial role in the body’s anti-infection defense. Due to the plasticity of the antigen-binding cleft, non-riboflavin antigens such as microbial molecules (49) and tumor cell-derived molecules (50) can bind to MR1, adding to the diversity of MR1 ligands. In bacterial and fungal infectious diseases, vitamin B2 metabolites produced are presented by MR1 to MAIT cells, which rapidly produce the pro-inflammatory factors IFN-γ, TNF, IL-17, and IL-22 (51). MAIT cells are activated by viral infections in an MR1-independent manner through innate cytokines and TCR receptors (52). In parasite research, MAIT cell activation correlates with parasite antigen concentration in human Malaria Infection (29). *Leishmania* does not encode riboflavin biosynthesis, but MAIT cells respond to *Leishmania* in a dose- and MR1-dependent manner and produce TNF, IFN-γ, and IL-17A to exert protection from *Leishmania* parasites (53). The 5µg/mL Emp stimulates high expression of CD155, a ligand for the TIGIT immunosuppressive receptor, in the HL-7702 hepatocyte cell line, mimicking T cell dysfunction due to TIGIT-CD155 interaction after *E.multilocularis* infection in an *in vitro* assay (13). Our results initially verified *in vitro* that MAIT cells have a tendency to increase in frequency after Emp stimulation. However, MAIT cells are constantly exposed to antigens (54) secreted from *E. multilocularis* or inflammatory signals produced by cytokines during *E. multilocularis* infections and further studies are needed to investigate the response and regulatory role of MAIT cells.

## 5 Conclusion

This study revealed differences in MAIT cell phenotype and function in the blood and liver of patients infected with *E. multilocularis*. The MAIT cells aggregated in liver tissue close to lesions exerted potential anti-AE effects through pro-inflammatory and pro-fibrotic functions. In addition, given the MAIT cells correlation with lesion size and response to surgical treatment, they have the potential to be biomarkers of disease progression. The present study expands our understanding of MAIT cells in parasitic infection diseases, and future studies should aim to further elucidate the mechanism of action of MAIT cells in AE diseases.

## Data availability statement

The data and material in the study are available from the corresponding author on reasonable request.

## Ethics statement

The studies involving humans were approved by the ethics committee of the First Affiliated Hospital of Xinjiang Medical University. The studies were conducted in accordance with the local legislation and institutional requirements. The participants provided their written informed consent to participate in this study.

## Author contributions

JL: Writing – review & editing, Writing – original draft, Visualization. HZ: Writing – review & editing, Resources. GL: Writing – review & editing, Validation, Supervision, Project administration, Methodology, Investigation. KA: Writing – review & editing, Supervision, Methodology, Investigation, Data curation. LL: Writing – review & editing, Validation, Software, Investigation, Formal analysis, Data curation. RL: Writing – review & editing, Supervision, Resources, Project administration. TA: Writing – review & editing, Supervision, Resources, Project administration, Funding acquisition.

## Supplementary material

Supplementary Figure 1
**(A)** Gating strategy of MAIT cells. Representative dot plots describing the percentage of MAIT cell among of T cells from AE patient. MAIT cells are defined as CD3^+^CD161^+^Vα7.2^+^ cells in the lymphocyte gate. **(B)** Representative dot plot showing CD161^+^Vα7.2^+^ cells and positive to 5-OP-RU loaded MR1 from AE patient’s PBMC. The quantities of CD3^+^CD161^+^Vα7.2^+^ and positive to 5-OP-RU loaded MR1 is no significant different between three different AE patients (*p*>0.05). **(C)** The MFI of CD28, CD69 and PD-1 (HD n=23-25; AE n=27-29) of MAIT cells. **(D)** The mean fluorescence intensity (MFI) of GzmB, IL-17A and IFN-γ (HD n=23-25; AE n=27-29) of MAIT cells. ns *p*>0.05; **p*<0.05; ***p*<0.01.

Supplementary Figure 2 Representative dot plots showing gating of surface receptor^+^ and cytokine^+^ MAIT cells.

Supplementary Figure 3
**(A)** Representative image of CD161^+^PD-1^+^ double positive cells in liver tissue sections from AE patients (n = 3), showing the presence of CD161^+^ PD-1^+^ cells in CLT more than DLT (Scale bar = 50 μm). **(B)** The MFI of cell surface receptors and secreted cytokines (n=8) of MAIT cells in AE patient liver tissues. **(C)** The degrees of fibrosis of AE patient’s circulating MAIT cell proportion predicted by APRI/FIB-4 evaluation (n = 6-12 per group). ns *p*>0.05; **p*<0.05; ***p*<0.01.

Supplementary Figure 4 Correlation of MAIT cell frequency with the percentage of lymphocyte, TBIL, IBIL, GGT, AST and ALT (n=28-29).

Supplementary Figure 5 Representative dot plots and data of the MAIT cell surface receptors and cytokines from PreOp and PostOp AE patients.

Supplementary Table 1 Specimens used for these studies. FCM, flow cytometry; IHC, immunohistochemistry; IF, immunofluorescence; α-SMA, alpha smooth muscle actin.

## References

[1] Wen H Vuitton L Tuxun T Li J Vuitton DA Zhang W. Echinococcosis: advances in the 21st century. Clin Microbiol Rev. (2019) 32:e00075–18. doi: 10.1128/cmr.00075-18 PMC6431127 30760475

[2] Torgerson PR Keller K Magnotta M and Ragland N. The global burden of alveolar echinococcosis. PloS Negl Trop Dis. (2010) 4:e722. doi: 10.1371/journal.pntd.0000722 20582310 PMC2889826

[3] Deplazes P Rinaldi L Alvarez Rojas CA Torgerson PR Harandi MF Romig T. Global distribution of alveolar and cystic echinococcosis. Adv Parasitol. (2017) 95:315–493. doi: 10.1016/bs.apar.2016.11.001 28131365

[4] Guo B Zhang Z Guo Y Guo G Wang H Ma J. High endemicity of alveolar echinococcosis in Yili Prefecture, Xinjiang Autonomous Region, the People's Republic of China: Infection status in different ethnic communities and in small mammals. PloS Negl Trop Dis. (2021) 15:e0008891. doi: 10.1371/journal.pntd.0008891 33465089 PMC7845998

[5] Salm LA Lachenmayer A Perrodin SF Candinas D and Beldi G. Surgical treatment strategies for hepatic alveolar echinococcosis. Food Waterborne Parasitol. (2019) 15:e00050. doi: 10.1016/j.fawpar.2019.e00050 32095621 PMC7034045

[6] Bhutani N Kajal P. Hepatic echinococcosis: A review. Ann Med Surg (Lond). (2018) 36:99–105. doi: 10.1016/j.amsu.2018.10.032 30450204 PMC6226561

[7] Wang H Zhang CS Fang BB Hou J Li WD Li ZD. Dual role of hepatic macrophages in the establishment of the *echinococcus multilocularis* metacestode in mice. Front Immunol. (2020) 11:600635. doi: 10.3389/fimmu.2020.600635 33488594 PMC7820908

[8] X B Huang H Gao S and Xu X. *Echinococcus multilocularis* induces surface high expression of inhibitory killer immunoglobulin-like receptor on natural killer cells. Allergol Immunopathol (Madr). (2021) 49:78–86. doi: 10.15586/aei.v49i5.465 34476926

[9] Wang J Cardoso R Marreros N Müller N Lundström-Stadelmann B Siffert M. Foxp3(+) T Regulatory Cells as a Potential Target for Immunotherapy against Primary Infection with *Echinococcus multilocularis* Eggs. Infection Immunity. (2018) 86:e00542-18. doi: 10.1128/iai.00542-18 PMC6204723 30037796

[10] Bakhtiar NM Spotin A Mahami-Oskouei M Ahmadpour E and Rostami A. Recent advances on innate immune pathways related to host-parasite cross-talk in cystic and alveolar echinococcosis. Parasit Vectors. (2020) 13:232. doi: 10.1186/s13071-020-04103-4 32375891 PMC7204293

[11] Wang J Jebbawi F Bellanger AP Beldi G Millon L and Gottstein B. Immunotherapy of *alveolar echinococcosis* via PD-1/PD-L1 immune checkpoint blockade in mice. Parasite Immunol. (2018) 40:e12596. doi: 10.1111/pim.12596 30315719 PMC6587932

[12] Jebbawi F Bellanger AP Lunström-Stadelmann B Rufener R Dosch M Goepfert C. Innate and adaptive immune responses following PD-L1 blockade in treating chronic murine *alveolar echinococcosis* . Parasite Immunol. (2021) 43:e12834. doi: 10.1111/pim.12834 33754355

[13] Zhang C Lin R Li Z Yang S Bi X Wang H. Immune exhaustion of T cells in *alveolar echinococcosis* patients and its reversal by blocking checkpoint receptor TIGIT in a murine model. Hepatology. (2020) 71:1297–315. doi: 10.1002/hep.30896 31410870

[14] Zhang C Wang H Li J Hou X Li L Wang W. Involvement of TIGIT in natural killer cell exhaustion and immune escape in patients and mouse model with liver *echinococcus multilocularis* infection. Hepatology. (2021) 74:3376–93. doi: 10.1002/hep.32035 34192365

[15] Godfrey DI Koay HF McCluskey J Gherardin NA. The biology and functional importance of MAIT cells. Nat Immunol. (2019) 20:1110–28. doi: 10.1038/s41590-019-0444-8 31406380

[16] Dusseaux M Martin E Serriari N Péguillet I Premel V Louis D. Human MAIT cells are xenobiotic-resistant, tissue-targeted, CD161hi IL-17-secreting T cells. Blood. (2011) 117:1250–9. doi: 10.1182/blood-2010-08-303339 21084709

[17] Gao MG Zhao XS. Mining the multifunction of mucosal-associated invariant T cells in hematological Malignancies and transplantation immunity: A promising hexagon soldier in immunomodulatory. Front Immunol. (2022) 13:931764. doi: 10.3389/fimmu.2022.931764 36052080 PMC9427077

[18] Wang H D'Souza C Lim XY Kostenko L Pediongco TJ Eckle SBG. MAIT cells protect against pulmonary *Legionella longbeachae* infection. Nat Commun. (2018) 9:3350. doi: 10.1038/s41467-018-05202-8 30135490 PMC6105587

[19] D'Souza C Pediongco T Wang H Scheerlinck JY Kostenko L Esterbauer R. Mucosal-associated invariant T cells augment immunopathology and gastritis in chronic *helicobacter pylori* infection. J Immunol. (2018) 200:1901–16. doi: 10.4049/jimmunol.1701512 29378910

[20] Parrot T Gorin JB Ponzetta A Maleki KT Kammann T Emgård J. MAIT cell activation and dynamics associated with COVID-19 disease severity. Sci Immunol. (2020) 5:eabe1670. doi: 10.1126/sciimmunol.abe1670 32989174 PMC7857393

[21] Beudeker BJB van Oord GW Arends JE Schulze Zur Wiesch J van der Heide MS de Knegt RJ. Mucosal-associated invariant T-cell frequency and function in blood and liver of HCV mono- and HCV/HIV co-infected patients with advanced fibrosis. Liver Int. (2018) 38:458–68. doi: 10.1111/liv.13544 PMC5836956 28792648

[22] Liu Y Zhu P Wang W Tan X Liu C Chen Y. Mucosal-associated invariant T cell dysregulation correlates with conjugated bilirubin level in chronic HBV infection. Hepatology. (2021) 73:1671–87. doi: 10.1002/hep.31602 33080074

[23] Zimmer CL Filipovic I Cornillet M O'Rourke CJ Berglin L Jansson H. Mucosal-associated invariant T-cell tumor infiltration predicts long-term survival in cholangiocarcinoma. Hepatology. (2022) 75:1154–68. doi: 10.1002/hep.32222 34719787

[24] Duan M Goswami S Shi JY Wu LJ Wang XY Ma JQ. Activated and exhausted MAIT cells foster disease progression and indicate poor outcome in hepatocellular carcinoma. Clin Cancer Res. (2019) 25:3304–16. doi: 10.1158/1078-0432.Ccr-18-3040 30723143

[25] Won EJ Ju JK Cho YN Jin HM Park KJ Kim TJ. Clinical relevance of circulating mucosal-associated invariant T cell levels and their anti-cancer activity in patients with mucosal-associated cancer. Oncotarget. (2016) 7:76274–90. doi: 10.18632/oncotarget.11187 PMC5342813 27517754

[26] von Seth E Zimmer CL Reuterwall-Hansson M Barakat A Arnelo U Bergquist A. Primary sclerosing cholangitis leads to dysfunction and loss of MAIT cells. Eur J Immunol. (2018) 48:1997–2004. doi: 10.1002/eji.201847608 30252934

[27] Atif M Warner S. and Oo YH Linking the gut and liver: crosstalk between regulatory T cells and mucosa-associated invariant T cells. Hepatol Int. (2018) 12:305–14. doi: 10.1007/s12072-018-9882-x PMC6097019 30027532

[28] Bolte FJ O'Keefe AC Webb LM Serti E Rivera E Liang TJ. Intra-hepatic depletion of mucosal-associated invariant T cells in hepatitis C virus-induced liver inflammation. Gastroenterology. (2017) 153:1392–1403.e2. doi: 10.1053/j.gastro.2017.07.043 28780074 PMC5669813

[29] Mpina M Maurice NJ Yajima M Slichter CK Miller HW Dutta M. Controlled human malaria infection leads to long-lasting changes in innate and innate-like lymphocyte populations. J Immunol. (2017) 199:107–18. doi: 10.4049/jimmunol.1601989 PMC5528886 28576979

[30] Jiang T Sun W Aji T Shao Y Guo C Zhang C. Single-cell heterogeneity of the liver-infiltrating lymphocytes in individuals with chronic *echinococcus multilocularis* infection. Infection Immunity. (2022) 90:e0017722. doi: 10.1128/iai.00177-22 36317875 PMC9670881

[31] Hegde P Weiss E Paradis V Wan J Mabire M Sukriti S. Mucosal-associated invariant T cells are a profibrogenic immune cell population in the liver. Nat Commun. (2018) 9:2146. doi: 10.1038/s41467-018-04450-y 29858567 PMC5984626

[32] Gottstein B Soboslay P Ortona E Wang J Siracusano A. and vuitton D immunology of alveolar and cystic echinococcosis (AE and CE). Adv Parasitol. (2017) 96:1–54. doi: 10.1016/bs.apar.2016.09.005 28212788

[33] Gherardin NA Souter MN Koay HF Mangas KM Seemann T Stinear TP. Human blood MAIT cell subsets defined using MR1 tetramers. Immunol Cell Biol. (2018) 96:507–25. doi: 10.1111/imcb.12021 PMC6446826 29437263

[34] Novak J Dobrovolny J Novakova L and Kozak T. The decrease in number and change in phenotype of mucosal-associated invariant T cells in the elderly and differences in men and women of reproductive age. Scand J Immunol. (2014) 80:271–5. doi: 10.1111/sji.12193 24846411

[35] Huang W He W Shi X Ye Q He X Dou L. Mucosal-associated invariant T-cells are severely reduced and exhausted in humans with chronic HBV infection. J Viral Hepat. (2020) 27:1096–107. doi: 10.1111/jvh.13341 32510704

[36] Dias J Hengst J Parrot T Leeansyah E Lunemann S Malone DFG. Chronic hepatitis delta virus infection leads to functional impairment and severe loss of MAIT cells. J Hepatol. (2019) 71:301–12. doi: 10.1016/j.jhep.2019.04.009 PMC6642010 31100314

[37] Yu H Yang A Liu L Mak JYW Fairlie DP and Cowley S. CXCL16 stimulates antigen-induced MAIT cell accumulation but trafficking during lung infection is CXCR6-independent. Front Immunol. (2020) 11:1773. doi: 10.3389/fimmu.2020.01773 32849637 PMC7426740

[38] Crotty S. T follicular helper cell biology: A decade of discovery and diseases. Immunity. (2019) 50:1132–48. doi: 10.1016/j.immuni.2019.04.011 PMC6532429 31117010

[39] Rao DA. T cells that help B cells in chronically inflamed tissues. Front Immunol. (2018) 9:1924. doi: 10.3389/fimmu.2018.01924 30190721 PMC6115497

[40] Li Y Tang L Guo L Chen C Gu S Zhou Y. CXCL13-mediated recruitment of intrahepatic CXCR5(+)CD8(+) T cells favors viral control in chronic HBV infection. J Hepatol. (2020) 72:420–30. doi: 10.1016/j.jhep.2019.09.031 31610223

[41] Vuitton DA. The ambiguous role of immunity in echinococcosis: protection of the host or of the parasite? Acta Trop. (2003) 85:119–32. doi: 10.1016/s0001-706x(02)00230-9 12606089

[42] Vuitton DA Zhang SL Yang Y Godot V Beurton I Mantion G. Survival strategy of *Echinococcus multilocularis* in the human host. Parasitol Int. (2006) 55 Suppl:S51–5. doi: 10.1016/j.parint.2005.11.007 16360335

[43] Mabire M Hegde P Hammoutene A Wan J Caër C Sayegh RA. MAIT cell inhibition promotes liver fibrosis regression *via* macrophage phenotype reprogramming. Nat Commun. (2023) 14:1830. doi: 10.1038/s41467-023-37453-5 37005415 PMC10067815

[44] Böttcher K Rombouts K Saffioti F Roccarina D Rosselli M Hall A. MAIT cells are chronically activated in patients with autoimmune liver disease and promote profibrogenic hepatic stellate cell activation. Hepatology. (2018) 68:172–86. doi: 10.1002/hep.29782 29328499

[45] Wang H Chen Z McCluskey J and Corbett AJ. Mouse models illuminate MAIT cell biology. Mol Immunol. (2021) 130:55–63. doi: 10.1016/j.molimm.2020.12.007 33360377 PMC7855494

[46] Le Bourhis L Martin E Péguillet I Guihot A Froux N Coré M. Antimicrobial activity of mucosal-associated invariant T cells. Nat Immunol. (2010) 11:701–8. doi: 10.1038/ni.1890 20581831

[47] Chua WJ Truscott SM Eickhoff CS Blazevic A Hoft DF and Hansen TH. Polyclonal mucosa-associated invariant T cells have unique innate functions in bacterial infection. Infection Immunity. (2012) 80:3256–67. doi: 10.1128/iai.00279-12 PMC3418730 22778103

[48] Grimaldi D Le Bourhis L Sauneuf B Dechartres A Rousseau C Ouaaz F. Specific MAIT cell behaviour among innate-like T lymphocytes in critically ill patients with severe infections. Intensive Care Med. (2014) 40:192–201. doi: 10.1007/s00134-013-3163-x 24322275

[49] Meermeier EW Laugel BF Sewell AK Corbett AJ Rossjohn J McCluskey J. Human TRAV1-2-negative MR1-restricted T cells detect S. pyogenes and alternatives to MAIT riboflavin-based antigens. Nat Commun. (2016) 7:12506. doi: 10.1038/ncomms12506 27527800 PMC4990709

[50] Lepore M Kalinichenko A Calogero S Kumar P Paleja B Schmaler M. Functionally diverse human T cells recognize non-microbial antigens presented by MR1. Elife. (2017) 6:e24476. doi: 10.7554/eLife.24476 28518056 PMC5459576

[51] Wong EB Ndung'u T and Kasprowicz VO. The role of mucosal-associated invariant T cells in infectious diseases. Immunology. (2017) 150:45–54. doi: 10.1111/imm.12673 27633333 PMC5341498

[52] van Wilgenburg B Scherwitzl I Hutchinson EC Leng T Kurioka A Kulicke C. MAIT cells are activated during human viral infections. Nat Commun. (2016) 7:11653. doi: 10.1038/ncomms11653 27337592 PMC4931007

[53] Moreira ML Borges-Fernandes LO Pascoal-Xavier MA Ribeiro ÁL Pereira VHS Pediongco T. The role of mucosal-associated invariant T cells in visceral leishmaniasis. Front Immunol. (2022) 13:926446. doi: 10.3389/fimmu.2022.926446 36189274 PMC9521739

[54] Valot B Rognon B Prenel A Baraquin A Knapp J Anelli M. Screening of antigenic vesicular fluid proteins of *Echinococcus multilocularis* as potential viability biomarkers to monitor drug response in alveolar echinococcosis patients. Proteomics Clin Appl. (2017) 11:11–12. doi: 10.1002/prca.201700010 28697272

