# Supplementary figures and images for: Phenotype and function of MAIT cells in patients with alveolar echinococcosis
Source: Front Immunol. 2024 Mar 14;15:1343567. doi: 10.3389/fimmu.2024.1343567 (PMC10973110; doi:10.3389/fimmu.2024.1343567)

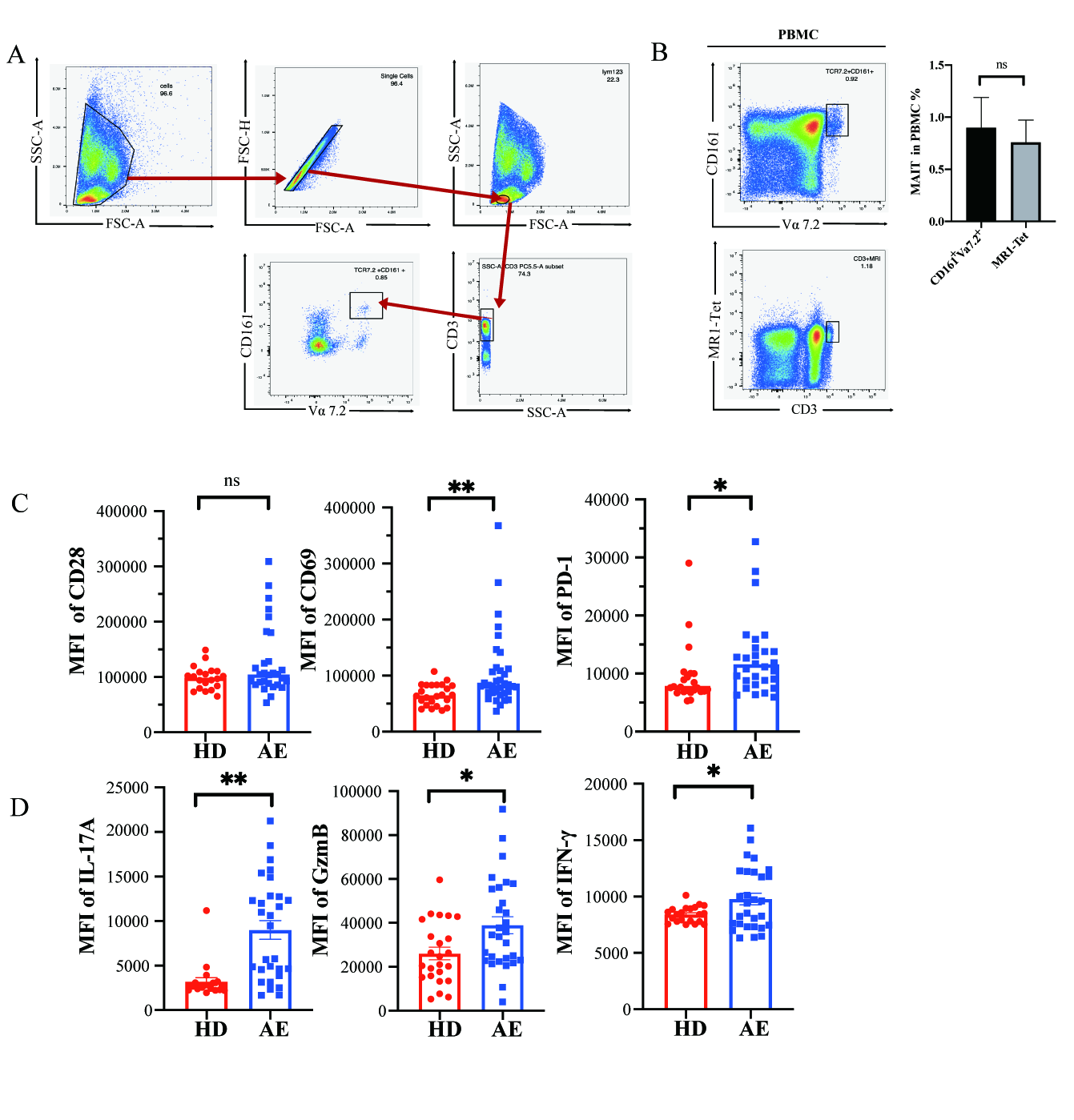

Supplement: Supplementary Figure 1 — (A) Gating strategy of MAIT cells. Representative dot plots describing the percentage of MAIT cell among of T cells from AE patient. MAIT cells are defined as CD3+CD161+Vα7.2+ cells in the lymphocyte gate. (B) Representative dot plot showing CD161+Vα7.2+ cells and positive to 5-OP-RU loaded MR1 from AE patient’s PBMC. The quantities of CD3+CD161+Vα7.2+ and positive to 5-OP-RU loaded MR1 is no significant different between three different AE patients (p>0.05). (C) The MFI of CD28, CD69 and PD-1 (HD n=23-25; AE n=27-29) of MAIT cells. (D) The mean fluorescence intensity (MFI) of GzmB, IL-17A and IFN-γ (HD n=23-25; AE n=27-29) of MAIT cells. ns p>0.05; *p<0.05; **p<0.01. [file Image_1.tif]

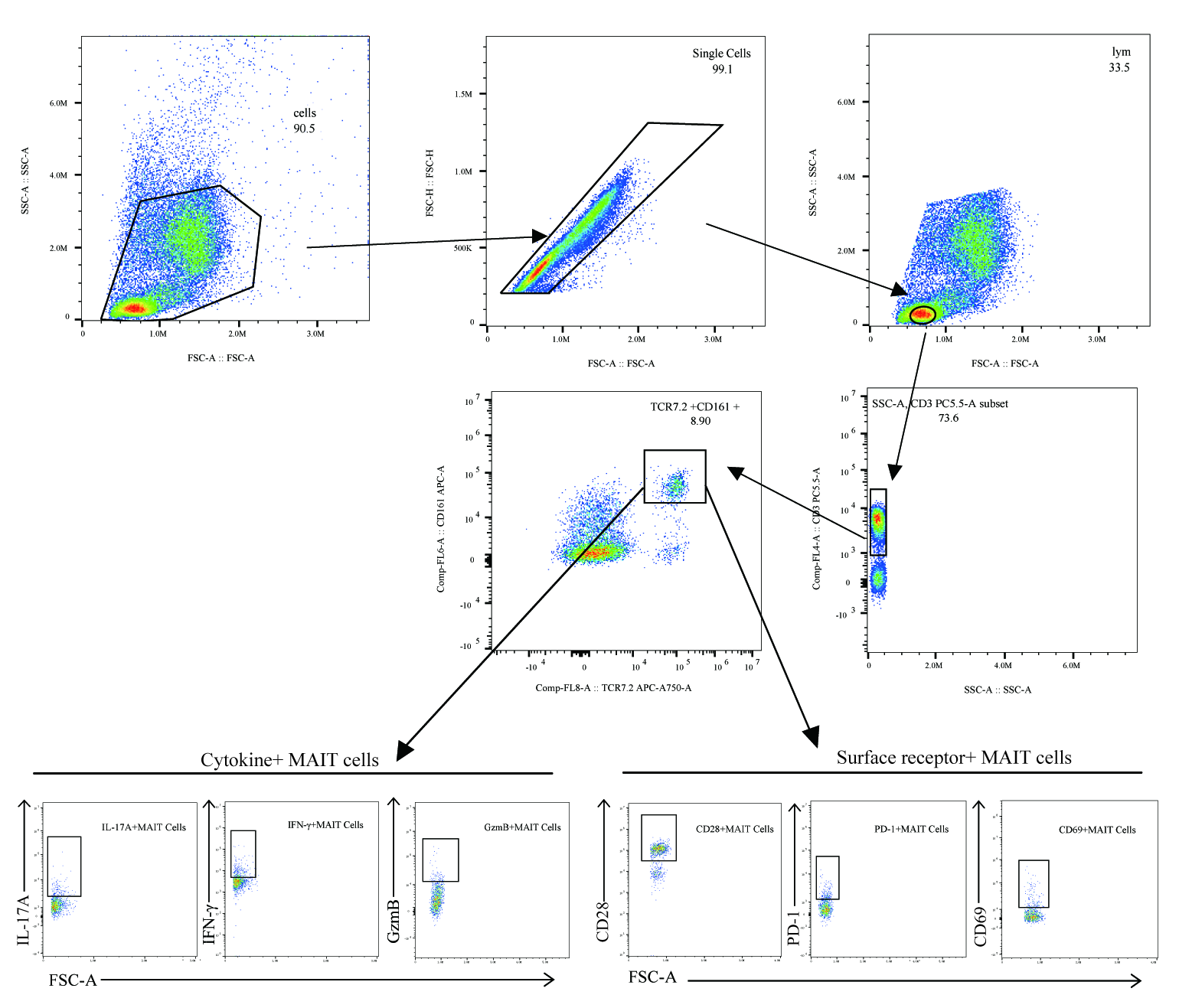

Supplement: Supplementary Figure 2 — Representative dot plots showing gating of surface receptor+ and cytokine+ MAIT cells. [file Image_2.tif]

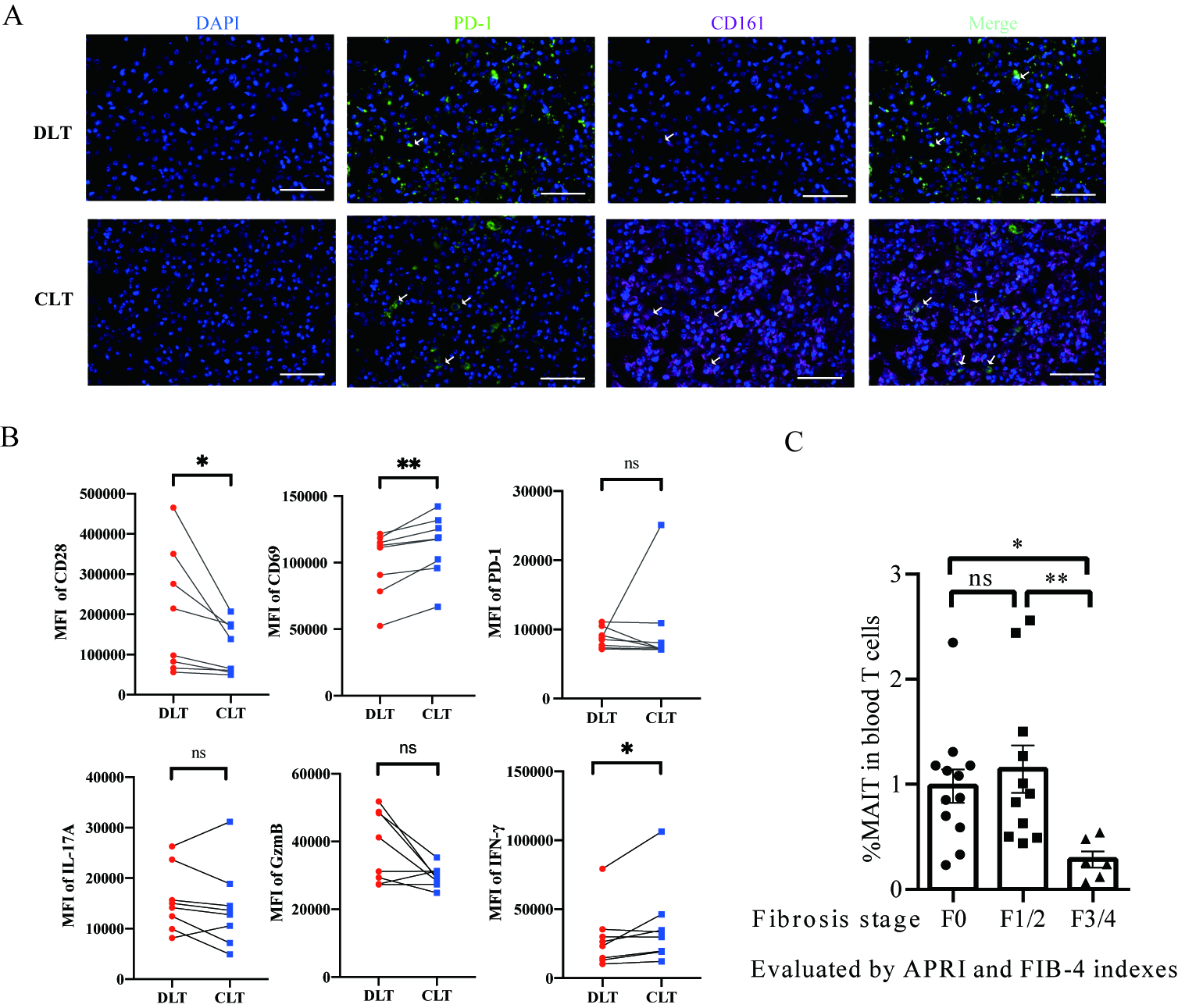

Supplement: Supplementary Figure 3 — (A) Representative image of CD161+PD-1+ double positive cells in liver tissue sections from AE patients (n = 3), showing the presence of CD161+ PD-1+ cells in CLT more than DLT (Scale bar = 50 μm). (B) The MFI of cell surface receptors and secreted cytokines (n=8) of MAIT cells in AE patient liver tissues. (C) The degrees of fibrosis of AE patient’s circulating MAIT cell proportion predicted by APRI/FIB-4 evaluation (n = 6-12 per group). ns p>0.05; *p<0.05; **p<0.01. [file Image_3.tif]

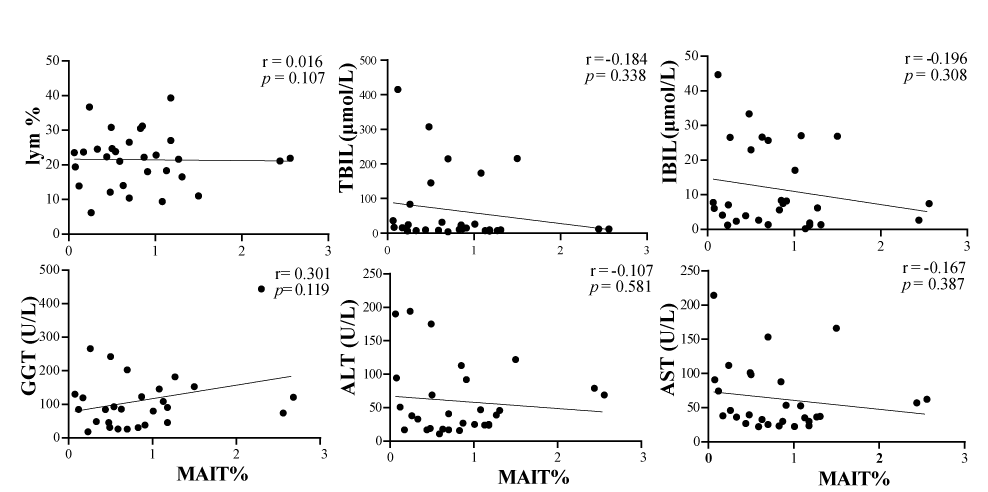

Supplement: Supplementary Figure 4 — Correlation of MAIT cell frequency with the percentage of lymphocyte, TBIL, IBIL, GGT, AST and ALT (n=28-29). [file Image_4.tif]

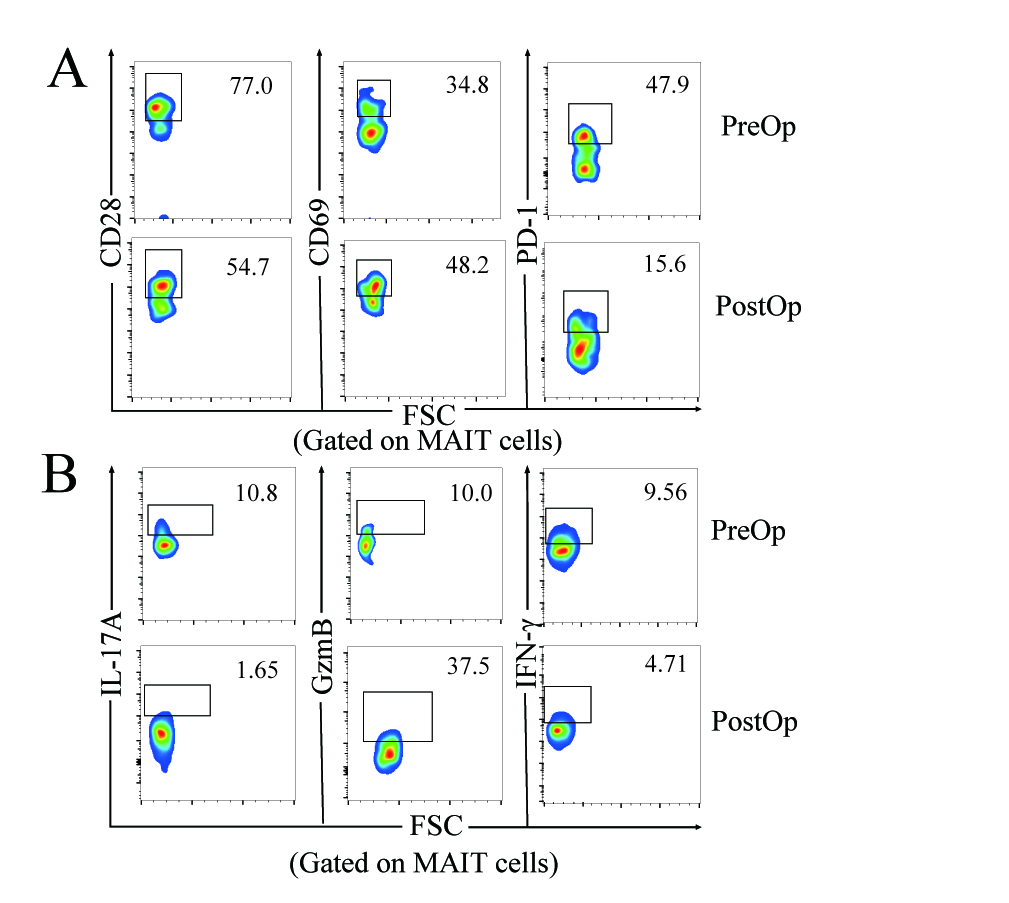

Supplement: Supplementary Figure 5 — Representative dot plots and data of the MAIT cell surface receptors and cytokines from PreOp and PostOp AE patients. [file Image_5.tif]
