# Supplementary material for: Phenotype and function of MAIT cells in patients with alveolar echinococcosis
Source: Front Immunol. 2024 Mar 14;15:1343567. doi: 10.3389/fimmu.2024.1343567 (PMC10973110; doi:10.3389/fimmu.2024.1343567)
Supplement: Supplementary Table 1 — Specimens used for these studies. FCM, flow cytometry; IHC, immunohistochemistry; IF, immunofluorescence; α-SMA, alpha smooth muscle actin. [file Table_1.docx]

**Supplementary table 1. Specimens used for these studies.**

| Experient | | The liver tissue of AE | | | | | | The blood of AE | | | The blood of HDs |
| --- | --- | --- | --- | --- | --- | --- | --- | --- | --- | --- | --- |
|  |  | CLT | | | DLT | Paired | | PreOp | PostOp | Paired |  |
| FCM | MAIT cells | | 10 | 10 | | | 10 | 29 | 8 | 8 | 25 |
|  | CD28, CD69, PD-1 | | 8 | 8 | | | 8 | 28-29 | 8 | 8 | 22-24 |
|  | IL-17A, GzmB, IFN-γ | | 8 | 8 | | | 8 | 23-29 | 8 | 8 | 14-24 |
| IHC | α-SMA | | 10 | 10 | | | 10 | - | - | - | - |
| IF | CD161, PD-1 | | 3 | 3 | | | 3 | - | - | - | - |

FCM, flow cytometry; IHC, immunohistochemistry; IF, immunofluorescence; α-SMA, alpha smooth muscle actin.
